# Replication of a pathogenic non-coding RNA increases DNA methylation in plants associated with a bromodomain-containing viroid-binding protein

**DOI:** 10.1038/srep35751

**Published:** 2016-10-21

**Authors:** Dian-Qiu Lv, Shang-Wu Liu, Jian-Hua Zhao, Bang-Jun Zhou, Shao-Peng Wang, Hui-Shan Guo, Yuan-Yuan Fang

**Affiliations:** 1 Virus-free Seedling Research Institute, Heilongjiang Academy of Agricultural Sciences, Harbin, China; 2 Heilongjiang Academy of Agricultural Sciences Postdoctoral Programme, Harbin, China.; 3 State Key Laboratory of Plant Genomics, Institute of Microbiology, Chinese Academy of Sciences, Beijing, China

## Abstract

Viroids are plant-pathogenic molecules made up of single-stranded circular non-coding RNAs. How replicating viroids interfere with host silencing remains largely unknown. In this study, we investigated the effects of a nuclear-replicating *Potato spindle tuber viroid* (PSTVd) on interference with plant RNA silencing. Using transient induction of silencing in *GFP* transgenic *Nicotiana benthamiana* plants (line 16c), we found that PSTVd replication accelerated *GFP* silencing and increased *Virp1* mRNA, which encodes bromodomain-containing viroid-binding protein 1 and is required for PSTVd replication. DNA methylation was increased in the *GFP* transgene promoter of PSTVd-replicating plants, indicating involvement of transcriptional gene silencing. Consistently, accelerated *GFP* silencing and increased DNA methylation in the of *GFP* transgene promoter were detected in plants transiently expressing *Virp1. Virp1* mRNA was also increased upon PSTVd infection in natural host potato plants. Reduced transcript levels of certain endogenous genes were also consistent with increases in DNA methylation in related gene promoters in PSTVd-infected potato plants. Together, our data demonstrate that PSTVd replication interferes with the nuclear silencing pathway in that host plant, and this is at least partially attributable to Virp1. This study provides new insights into the plant-viroid interaction on viroid pathogenicity by subverting the plant cell silencing machinery.

Many eukaryotes share highly abundant classes of small RNAs (sRNAs) that range in size from 20 to 25 nucleotides (nt) produced from longer double-stranded RNA (dsRNA) precursors through the action of RNase III-like Dicer or Dicer-like (DCL) enzymes^1^,^2^. The sRNAs participate in RNA silencing (or RNA interference, RNAi) by binding to an ARGONAUTE (AGO) protein in RNA-induced silencing complexes (RISCs) and act as sequence-specificity determinants through RNA-RNA and also RNA-DNA interactions. These interactions mediate a wide range of phenomena such as post-transcriptional gene silencing (PTGS) of mRNA and transcriptional silencing (TGS) of heterochromatin, as well as translational inhibition of mRNA^3^,^4^,^5^. In plants, the TGS pathway consists of RNA-directed DNA methylation (RdDM), which requires both 24-nt small interfering RNAs (siRNAs) and long non-coding RNA transcripts as well as proteins, such as DNA-dependent RNA polymerase IV (Pol IV), RNA-dependent RNA polymerase 2 (RDR2) and AGO4 for de novo DNA methylation. This pathway has a great impact on the expression and constitution of the plant genome^5^.

In addition to regulation of genome integrity and development, RNAi has roles in transposon restriction and anti-pathogen defense^1^,^6^,^7^,^8^,^9^. As a counterstrategy to host RNA silencing, many viruses encode suppressor proteins (VSR) targeting DCL, RISC or small RNA activities^10^,^11^,^12^,^13^,^14^. sRNAs derived from some subviruses, such as viroids and viral satellites, were also detected in infected plants, suggesting that these unconventional RNAs are also targeted by PTGS^15^,^16^,^17^,^18^,^19^,^20^,^21^,^22^,^23^,^24^. However, these subviruses may possess novel silencing suppressor activities that counter-defend against host silencing. The function of these subviruese is attributed to the resistance of their secondary structures to RISC-mediated cleavage rather than directly targeting the host silencing components as some VSRs do^25^,^26^.

Viroids are the smallest of transmissible molecules, ranging from 246 to 401 nucleotides in size^27^, and are made up of single-stranded circular RNA that is non-translatable and autonomously replicates by redirected host processes^27^,^28^. A type member of the *Pospiviroidae* viroid family, *Potato spindle tuber viroid* (PSTVd) replicates in the nucleus via an asymmetric rolling circle mechanism with the aid of host-encoded DNA-dependent RNA polymerase II^28^,^29^,^30^,^31^. A host RNA-binding protein, viroid-binding protein 1 (Virp1), was shown to interact with PSTVd and was required for PSTVd infection in both *Nicotiana* plants and protoplasts^32^,^33^. Early work by Wassenegger and colleagues on viroid-host interactions led to the landmark discovery of RNA-directed DNA methylation (RdDM). These researchers introduced PSTVd cDNA into the tobacco genome and found that the autonomous viroid RNA-RNA replication triggered viroid cDNA methylation^34^.

It has recently been reported that infection of cucumbers with a nuclear-replicating Hop stunt viroid (HSVd) is accompanied by changes in DNA methylation of host ribosomal RNA genes^35^. However, the molecular basis of nuclear-replicating viroid interference with host silencing pathways remains largely unknown. In this study, we investigated the effects of PSTVd replication on induction of a transgene *GFP* silencing in *N. benthamiana* and found that PSTVd replication accelerated, rather than suppressed, *GFP* silencing in the transient expression system in the 16c GFP plant. Consistently, DNA methylation was increased in the *GFP* transgene promoter of PSTVd-infected 16c plants and was associated with Virp1, whose coding mRNA was induced by PSTVd replication. We also found that *Virp1* mRNA was increased upon PSTVd infection in natural host potato plants. Reduced transcription of certain endogenous genes was also consistent with the increases in DNA methylation in related gene promoters in PSTVd-infected plants in which accumulation of tested endogenous miRNAs was not affected. Together, our results demonstrate that the host Viroid-binding bromodomain-containing protein, Virp1, which is required and induced by PSTVd infection, may partially contribute to the interference of the nuclear silencing pathway in PSTVd-infected plants.

## Materials and Methods

### Plant Materials and Growth Conditions

The GFP-transgenic *Nicotiana benthamiana* (line 16C) was described in previous study^13^. In this system, an *Agrobacterium* strain expressing 35S-GFP that is infiltrated to 16c plants induced silencing of the *GFP* transgene, resulting in red fluorescence. The cultivar *Solanum tuberosum* (KEXIN NO. 18) and *Nicotiana benthamiana* plants were grown in a glass house at 25 °C with16-h-light/8-h-dark cycles.

### Cloning and Constructs

For the 35S-PSTVd construct, full-length PSTVd cDNAs were amplified by RT-PCR using total RNAs extracted from PSTVd infected potato leaves as the template. The RT-PCR products were cloned into T-Vectors (Tsingke) using T4 DNA Ligase (Thermo). After confirmation by sequencing, two full-length minus strand PSTVd cDNAs were tandemly linked and subcloned into the pCAMBIA1300-221 binary vectors. For 35S-∆PSTVd construct, the 340-bp full-length minus strand PSTVd RT-PCR product in T-Vectors was subcloned into the pCAMBIA1300-221 binary vectors. Replicative 35S-PSTVd and non-replicative 35S-∆PSTVd were confirmed using transient expression system in *N. benthamiana* and RNA gel blotting analysis (Supplementary Figure S1).

For the 35S-Virp1 construct, the 1848-bp sequence of the *Virp1* gene was amplified from *N. benthamiana*, and the RT-PCR products were cloned into T-Vectors (Tsingke) using T4 DNA Ligase (Thermo). After confirmation by sequencing, *NbVirp1* sequence was subcloned into the pCAMBIA1300-221 binary vectors. The *NbVirp1* and *Virp1* from *Solanum tuberosum (StVirp1*) sequences share 87% identity at the nucleotide level (Supplementary Figure S2).

For the Virp1i construct, the 158bp sense and antisense sequences of the middle part of the bromodomain of *NbVirp1* were amplified, and the two PCR fragments were each cloned into T-vectors (Tsingke) using T4 DNA Ligase (Thermo). After confirmation by sequencing, the two sequences were inserted into an intron-containing intermediate construct (pSK-int)^36^, to obtain sequence cassettes containing the inverted-repeat RNAi constructs as previously described^36^, producing pSK-Virp1i. The cassettes were then subcloned into the pCAMBIA1300-221 binary vector to generate Virp1i.

A diagram of all constructs used in this study is shown in Supplementary Figure S3.

### Viroid Inoculation and Transient Expression

Two-week-old *Solanum tuberosum* were inoculated with sap prepared freshly from wild-type PSTVd infected plants by rubbing mechanically. The phosphate buffer inoculated leaves were used as mock.

For transient assays, the EHA105 strain of *Agrobacterium tumefaciens* was transformed with the 35S-GFP, 35S-PSTVd, 35S-∆PSTVd, 35S-Virp1, 35S-Virp1i, 35S-GUS or 35S-P19 by electroporation, and transformants were selected on Luria-Bertani medium containing rifampicin at 10 mg/liter and kanamycin at 50 mg/liter. Equal volumes of Agrobacterium cultures containing 35S-GFP (OD600 1.0) with either 35S-PSTVd, 35S-∆PSTVd, 35S-Virp1 or 35S-P19, 35S-GUS control (OD600 1.5) were mixed and co-infiltrated into 5-week-old 16c leaves. For Virp1i infiltration, Agrobacterium cultures containing Virp1i (OD600 1.0) were infiltrated into 16c leaves. The endogenous *NbVirp1* was silenced at 3dpi, and the silenced leaves were then used for the co-infiltration assay.

### RNA Extraction and RNA Gel Blot Analysis

Total RNA was extracted from *Agrobacterium*-infiltrated *N. benthamiana* or viroid inoculated *S. tuberosum* leaf tissues using the hot-phenol method as previously described^13^. For high molecular weight RNA gel blots, 15 μg of total RNA was separated on 1.2% agarose gels containing 6% formaldehyde and transferred to nylon N^+^ membrane. For mature PSTVd and siPSTVd detection, the [a-^32^P]-UTP-labelled T7 RNA transcripts from cDNA clones in pGEM-Teasy vector using the MAXIscriptkit (Ambion) were used as probes; for GFP mRNA detection, the full length GFP were radioactively labelled by [a-^32^P]-dCTP using a Rediprime™II Random Prime Labelling System(GE healthcare), for siGFP detection, the [a-^32^P]-UTP-labelled T7 RNA transcripts from pGEM-Teasy-GFP vector using the MAXIscriptkit (Ambion) were used as probes; for *Virp1* mRNA detection, the full length NbVirp1 were radioactively labelled by [a-^32^P]-dCTP using a Rediprime™II Random Prime Labelling System(GE healthcare); for miR167, miR159 and U6 detection, [r-^32^P]ATP-labeled specific oligonucleotide probe sequences were used.

Dot-blot hybridization detection of PSTVd accumulation was performed as described (Monger WA *et al*. 2015). The PSTVd DNA fragment was labeled using the DIG DNA PCR labeling kit (Mylab Corporation).

### DNA Bisulfite Sequencing Analysis

DNA bisulfite sequencing analysis: incubation of the target DNA with sodium bisulfite results in conversion of unmethylated cytosine residues into uracil, leaving the methylated cytosines unchanged. Total DNA was extracted using the DNeasy Plant Mini Kit (Qiagen). A total of 1μg of DNA was used for bisulfate treatment using the EpiTect Bisulfite kit (Qiagen). The purified bisulfite-treated DNA was amplified, ligated into the pGEM-T vector (Tsingke), and sequenced. The cytosine methylation analysis was performed via the CyMATE program (http://www.cymate.org/).

### Quantitative RT-PCR

Total RNA was extracted from *Solanum tuberosum* leaves using TRIzol reagent (Thermo). Genomic DNA was digested using DNase I (Takara) and then reverse-transcribed into cDNA using the GoScript™ Reverse Transcription System (Promega). qRT-PCR analysis was performed with a 1000 series Thermal Cycling Platform (Bio-Rad) using EvaGreen 2X qPCR MasterMIX (Applied Biological Materials Inc.). At least three biological replicates and three technical replicates within an experiment for each sample were performed.

### McrBC Digestion-PCR

McrBC is an endonuclease which cleaves DNA containing methylcytosine on one or both strands, McrBC will not act upon unmethylated DNA. *S. tuberosum* genomic DNA was extracted using the DNeasy Plant Mini Kit (Qiagen). A total of 3 μg of genomic DNA was digested by McrBC (Biolabs) at 37 °C overnight, precipitated by ethanol, and dissolved with nuclease-free water. A total of 40 ng McrBC-digested DNA was used for each PCR reaction.

All primer and probe sequences are listed in Supplementary Table S1.

## Results

### PSTVd accelerated *GFP* silencing in transient assays

**Figure 1:**
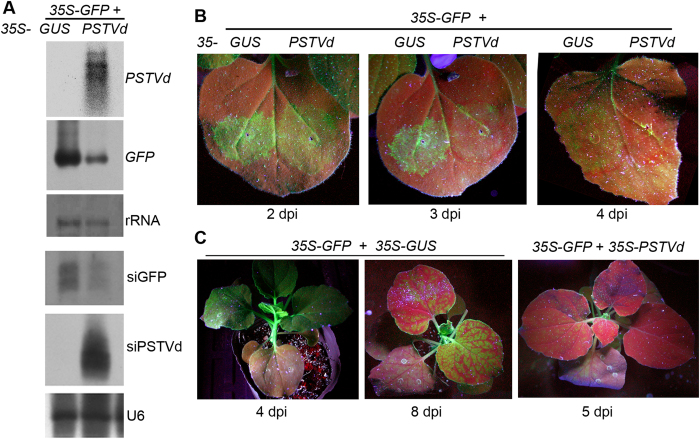
Effects of PSTVd replication on *GFP* silencing in transient assays. (**A**) RNA gel blot detection of mature PSTVd RNA, *GFP* mRNA, PSTVd-derived siRNA (siPSTVd) and GFP-derived siRNA (siGFP) accumulation in 16c plants co-infiltrated with *35S-GFP* and either *35S-GUS* control or *35S-PSTVd* at 4 dpi. Methylene blue-stained ribosome rRNA and U6 hybridization were used as the loading control. (**B**) Observation of local *GFP* silencing in the leaves of 16c plants co-infiltrated with *35S-GFP* and either *35S-GUS* control or *35S-PSTVd*. Photographs were taken under UV light at 2, 3 and 4 dpi. (**C**) Observation of systemic *GFP* silencing spread to the whole plant induced by co-infiltrated *35S-GFP* with either *35S-GUS* control or *35S-PSTVd* in separate plants. Photographs were taken under UV light at 4, 5 and 8 dpi.

To investigate the effects of PSTVd replication on interference with plant RNA silencing processes, we first created a construct consisting of two tandem linked full-length minus strand PSTVd cDNAs under the control of the 35S promoter, resulting in *35S-PSTVd*. Autonomous replication of the PSTVd RNA was confirmed by detection of mature PSTVd RNA in transient expression of *35S-PSTVd* in the leaves of 16c using an Agro-infiltration assay (Fig. 1A). To test the effect on RNA silencing, *Agrobaterium* strains expressing *35S-PSTVd* and *35S-GFP* were co-infiltrated into the leaves of 16c plants. To our surprise, co-infiltration with *35S-PSTVd* resulted in a significant low-intensity GFP green fluorescence in the infiltrated areas (Fig. 1B), compared to co-infiltration of *35S-GFP* with the *35S-GUS* control in the same leaves at 2 days post-infiltration (dpi) (Fig. 1B), where the GFP green fluorescence was maintained up to 3 dpi and began to decrease at 4 dpi (Fig. 1B). Red fluorescence appeared in areas co-infiltrated with *35S-GFP*/*35S-PSTVd*, indicating silencing of the *GFP* transgene (Fig. 1B).

To better observe GFP systemic silencing, we co-infiltrated *35S-GFP* with *35S-PSTVd* or *35S-GUS* in separate plants. All plants co-infiltrated with *35S-PSTVd* displayed systemic GFP silencing at 3 dpi and almost complete silencing at 5 dpi (Fig. 1C). At this point in plants co-infiltrated with *35S-GFP*/*35S-GUS*, GFP silencing was only found in some infiltrated areas (Fig. 1C at 4 dpi), while systemic silencing occurred at 6–8 dpi (Fig. 1C). Similar results were observed in three independent experiments in which a total of 36 plants were used for each infiltration combination. Observation of both local and systemic *GFP* silencing suggested that co-infiltration with *35S-PSTVd* might accelerate rather than suppress *GFP* silencing in the transient assay system.

Infiltrated leaves at 4 dpi were collected for total RNA and small RNA extraction for RNA gel blotting analysis. Large amounts of the siRNA derived from PSTVd (siPSTVd) were detected in leaves co-infiltrated with *35S-PSTVd* (Fig. 1A), consistent with previous reports that viroid RNAs can trigger RNA silencing^15^,^26^. The *GFP* mRNA in leaves co-infiltrated with *35S-PSTVd* accumulated at lower levels than those co-infiltrated with *35S-GUS*, consistent with the low-intensity GFP fluorescence observed in *35S-PSTVd* co-infiltrated leaves. However, the small RNA of GFP (siGFP) was detected with a significantly lower accumulation level in leaves co-infiltrated with *35S-PSTVd* compared to leaves co-infiltrated with *35S-GUS* (Fig. 1A). This finding contradicted the prevalent view that lower accumulation of *GFP* mRNA is normally accompanied by higher *GFP*-derived siRNA accumulation in the transient assay. Our result suggested that the low accumulation level of *GFP* mRNA did not entirely result from PTGS-mediated degradation of *GFP* mRNA that produced siGFP and implied that nuclear-replicating PSTVd may interfere with the expression of *GFP* mRNA at the transcriptional level.

### PSTVd-induced host viroid-binding protein played a role in the acceleration of *GFP* silencing

**Figure 2:**
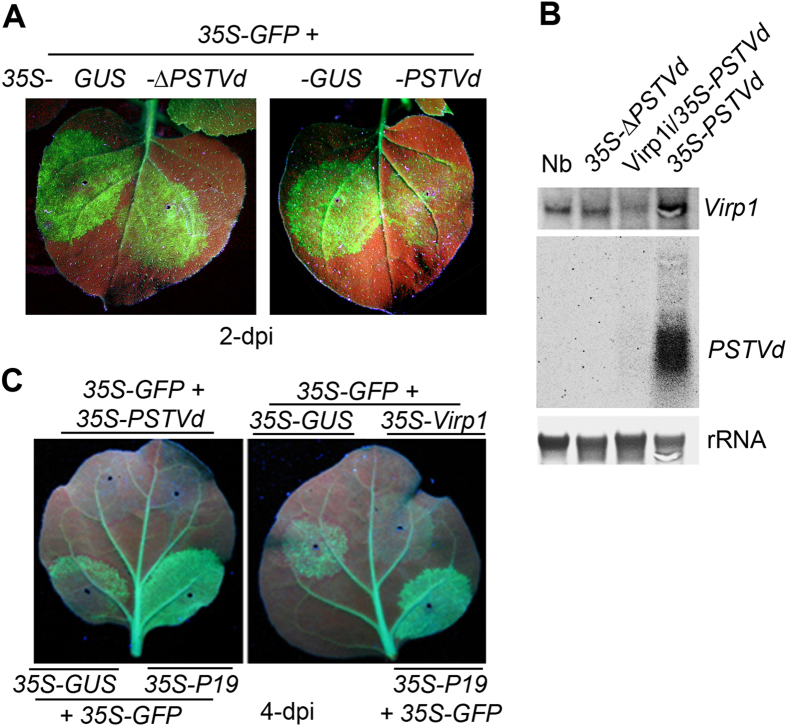
Effects of PSTVd-induced host *Virp1* on the acceleration of *GFP* silencing in transient assays. (**A**) GFP fluorescence in the leaves of 16c plants co-infiltrated with *35S-GFP* and either *35S- GUS* control or *35S-PSTVd* or *35S-∆PSTVd.* Photographs were taken under UV light at 2 dpi. (**B**) RNA gel blot detection of *Virp1* mRNA and mature PSTVd RNA accumulation in 16c plants and *Virp1* silencing leaves co-infiltrated with *35S-GFP* and either *35S-GUS* control or *35S-PSTVd* or *35S-∆PSTVd* at 4 dpi. Methylene blue-stained ribosome rRNA was used as the loading control. (**C**) GFP fluorescence in leaves of 16c plants co-infiltrated with *35S-GFP* and either *35S-GUS, 35S-P19* control or *35S-PSTVd* or *35S-Virp1.* Photographs were taken under UV light at 4 dpi.

To detect whether the 35S promoter-derived replication of the PSTVd sequence or the replicating PSTVd viroid caused the enhancement of *GFP* silencing, a non-replicative full-length minus strand PSTVd cDNA under the 35S promoter was constructed, resulting in *35S-ΔPSTVd*. 16c plants were co-infiltrated with *35S-GFP* and*35S-GUS, 35S-PSTVd* or *35S-ΔPSTVd*. An intense GFP green fluorescence area was observed after *35S-GFP* co-infiltration with *35S-GUS* or *35S-∆PSTVd* compared to areas co-infiltrated with *35S-PSTVd* (Fig. 2A), indicating that replication of PSTVd was required for the enhancement of *GFP* silencing. Previous studies showed that PSTVd interacts with the host RNA-binding protein, Virp1^32^,^33^. We therefore examined the response of *Virp1* upon PSTVd replication in 16c plants. Increased accumulation of *Virp1* mRNA was detected when *35S-PSTVd* was infiltrated into 16c plants leaves compared to non-infiltrated 16c leaves with or without *35S-∆PSTVd* infiltration (Fig. 2B). Replication of PSTVd was confirmed in the 35S-PSTVd-infiltrated leaves (Fig. 2B). Silencing of *Virp1* in 16c leaves by infiltration of an RNAi construct, Virp1i, restricted PSTVd replication (Fig. 2B), which was consistent with the early finding that *Virp1* was required for PSTVd infection in *Nicotiana* plants^33^. We then examined the effect of *Virp1* on the PSTVd-accelerated *GFP* silencing process. Co-infiltration of *35S-GFP* with *35S-PSTVd* or *35S-Virp1* was then performed in 16c plants. Co-infiltration of *35S-GFP* with *35S-GUS* or *35S-P19*, a viral suppressor protein, was used the control. The GFP green fluorescence rapidly decreased by 4 dpi after co-infiltration with *35S-PSTVd* or *35S-Virp1*, whereas green fluorescence was maintained after co-infiltration with controls at this time point (Fig. 2C). This result demonstrates that the reduced *GFP* mRNA level (Fig. 1A) is not attributable to replicative competition by the propagative PSTVd and also suggests that *Virp1*, which was induced by propagative PSTVd played at least a partial role in this enhanced silencing process.

### PSTVd replication, Virp1 reduced expression and increased DNA methylation of the *GFP* transgene

**Figure 3:**
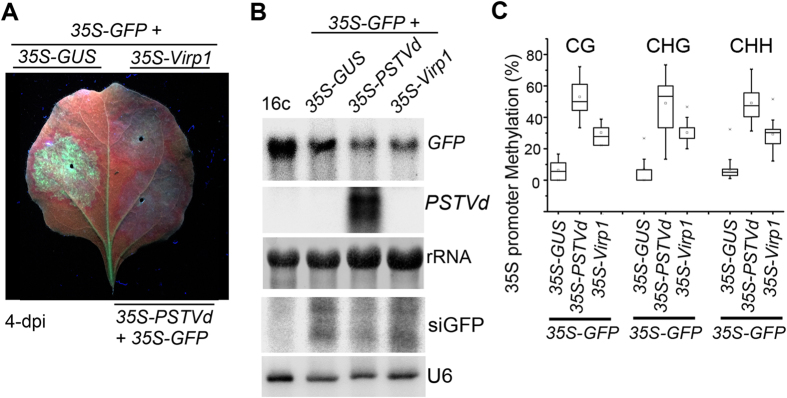
Effects of PSTVd replication and Virp1 on the expression and DNA methylation of the *GFP* transgene. (**A**) GFP fluorescence in leaves of 16c plants co-infiltrated with *35S-GFP* and either *35S-GUS* control, 3*5S-PSTVd* or *35S-Virp1.* Photographs were taken under UV light at 4 dpi. (**B**) RNA gel blot detection of *GFP* mRNA, mature PSTVd RNA and siGFP accumulation from samples as described in (**A**). Methylene blue-stained ribosome rRNA and U6 hybridization were used as the loading control. (**C**) Percentage of CG, CHG, and CHH methylation of the 35S promoter of the *35S-GFP* transgene by bisulfite sequencing analysis from samples as described in (**A**). The original data are shown in the Supplementary Figure S4. The statistical analysis was performed using the OriginPro 8 program.

We then examined the accumulation level of *GFP* mRNA after co-infiltration of *35S-GFP* with *35S-Virp1*. Total RNA was isolated from co-infiltrated leaves of the 16c plant. The accumulation of *GFP* mRNA was reduced in leaves co-infiltrated with *35S-GUS* at 4-dpi, consistent with the induction of *GFP* silencing with a red ring observed (Fig. 3A,B). Notably, the *GFP* mRNA level was even lower in leaves co-infiltrated with *35S-PSTVd* or *35S-Virp1* compared to leaves co-infiltrated with *35S-GUS* at this time point (Fig. 3B). This result demonstrates that the PSTVd replication increased silencing of *GFP* mRNA was, at least in part, attributable to the functions of Virp1, whose mRNA was induced by PSTVd replication (Fig. 2). Low levels of siGFP were again detected in leaves co-infiltrated with *35S-GFP*/*35S-PSTVd* but not in *35S-GFP*/*35S-Virp1* compared to control *35S-GFP*/*35S-GUS* co-infiltration (Fig. 3B). These results suggest that, in addition to the degradation at the PTGS level, the enhanced silencing of *GFP* by replicative PSTVd or Virp1 was reinforced at the TGS level by altering the *GFP* mRNA expression level.

As DNA methylation at the promoter sequences can cause TGS^20^, we investigated if DNA methylation occurred in the 35S promoter of the *35S-GFP* transgene using bisulfite sequencing. DNA samples were extracted from leaves co-infiltrated with *35S-GFP* and *35S-PSTVd, 35S-Virp1* or *35S-GUS*. As summarized in Fig. 3C, the sequencing results revealed increases in methylation of CG, CHG and CHH in the 35S promoter region of *35S-PSTVd*-co-infiltrated leaves compared to the*35S-GUS*-co-infiltrated leaves. In the *35S-Virp1*-co-infiltrated leaves, an increased ratio of DNA methylation was also detected compared to that of *35S-GUS*-co-infiltrated leaves, demonstrating that expression of *Virp1* also contributed to the changes of DNA methylation. The increased ratio of DNA methylation in *35S-PSTVd*-co-infiltrated leaves was clearly higher than in *35S-Virp1*-co-infiltrated leaves, suggesting that, in addition to Virp1, other factor(s) may also be involved in the increases in DNA methylation during PSTVd replication in plants.

Taken together, our data demonstrate that PSTVd replication caused the reduced expression of the *GFP* transgene and the increased DNA methylation of its promoter, a process that is partially dependent on the functions of *Virp1*, which is increased upon replication of PSTVd.

### PSTVd infection induced *Virp1* mRNA and increased DNA methylation in host potato plants

**Figure 4:**
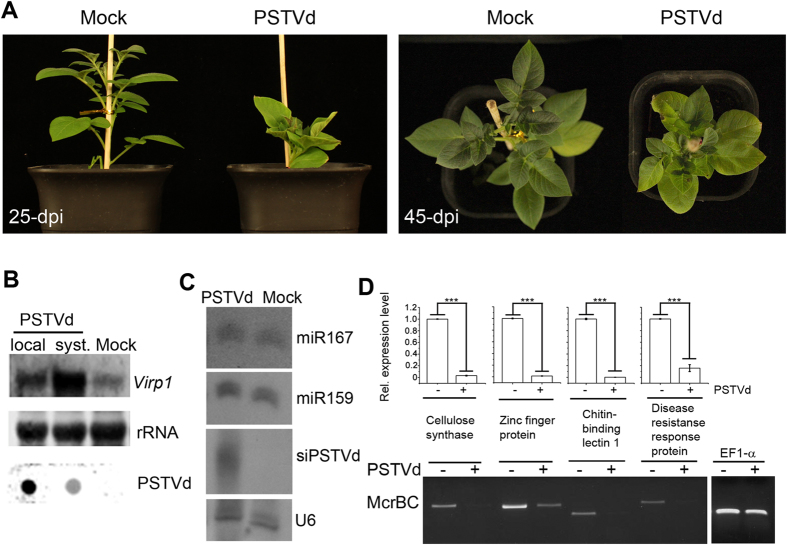
Effects of PSTVd infection on *Virp1* expression and DNA methylation in host potato plants. (**A**) Disease symptoms on potato plants inoculated with PSTVd at 25-dpi and 45-dpi. (**B**) RNA gel blot detection of *Virp1* mRNA accumulation and Dot-blot hybridization detection of PSTVd accumulation in both inoculated and systemic leaves of the host potato plants. Methylene blue-stained ribosome rRNA was used as the loading control. (**C**) RNA gel blot analysis of endogenous small RNAs and siPSTVd in PSTVd-infected host potato plants. U6 hybridization was used as the loading control. (**D**) Quantitative RT-PCR analysis of the expression levels of the selected genes in PSTVd-infected host potato plants. Error bars represent SD for three replicates. Relative transcript levels were calculated by the ∆∆C(t) method (Livak and Schmittgen, 2001) using EF1-a transcripts (potato elongation factor 1−α) as the internal standard. The value of mRNA in plants (−) was arbitrarily designated as 1. The asterisks indicate significant differences (P < 0.05, one-way ANOVA). McrBC PCR analysis of the DNA methylation levels of selected gene promoter sequences in PSTVd-infected host potato plants.

Next, we investigated whether PSTVd infection in natural host potato induced *Virp1* mRNA. Potato plants were inoculated with sap extracted from natural PSTVd-infected potato plants. Typical symptoms were observed in infected plants with a decrease in shoot and internode length, shortening of petioles and distortion of leaves (Fig. 4A). Replication of PSTVd in infected potato was detected in both inoculated and systemic leaves (Fig. 4B). The high level of viroid detected in inoculated leaves probably included the initial inoculums. The*Virp1* mRNA expression level was induced in both inoculated and, especially, systemic leaves (Fig. 4B). This result demonstrates that PSTVd infection in the host potato plants also greatly increased *Virp1* mRNA expression. microRNAs are known to control the expression of genes involved in several developmental processes. To detect whether PSTVd infection affects the host miRNA pathway, the accumulation of conserved miRNAs was examined. There were no significant differences in miR159 and miR167 accumulation in potato plants before and after PSTVd infection (Fig. 4C). siPSTVd was detected in PSTVd-infected potato plants (Fig. 4C). These data demonstrate that replication of viroid RNA in potato also triggers RNA silencing and greatly induces *Virp1* mRNA. PSTVd infection and increases in *Virp1* transcripts do not significantly affect the miRNA pathway in potato plants.

We then examined whether PSTVd-infected potato would have an effect on DNA methylation. Four sequenced genes with a high cytosine context in promoters were selected to examine expression levels and DNA methylation. Quantitative RT-PCR analysis demonstrated that the expression of the selected genes decreased in PSTVd-infected potato plants compared to the mock inoculation wild-type plants (Fig. 4D). We then investigated the methylation state of the selected gene promoter sequences using McrBC digestion-PCR. McrBC is a methylation-dependent restriction enzyme that recognizes DNA containing two or more methylated cytosine residues and cleaves the DNA at multiple sites close to one of the methylated cytosines. PCR-amplified McrBC-digested DNA resulted in a clear reduction in the amplification of four tested gene promoters in PSTVd-infected potato plants, whereas there was no significant difference in the amplification of the EF1-α gene promoter which contains a low level of cytosine residues (Fig. 4D). This indicated that the tested gene promoters were hypomethylated in PSTVd-infected plants. Taken together, consistent with the finding of PSTVd-induced Virp1-associated expediting *GFP* silencing in *N. benthamiana* plants, PSTVd infection in natural host potato plants also increased *Virp1* transcript and DNA methylation in promoters of certain endogenous genes resulting in reduced transcription levels.

## Discussion

In this study, we showed that PSTVd infection in *N. benthamiana* and its natural host potato plants triggered RNA silencing and produced a large amount of siPSTVd. However, unlike many viruses encoding suppressor proteins (VSR) to counter host anti-viral RNA silencing, we found that replication of PSTVd accelerated *GFP* silencing in a transient expression assay by increasing TGS via enhancing target promoter DNA methylation. The Virp1 coding gene was required for and induced by PSTVd replication and, in part, contributed to promoter methylation of the *GFP* transgene. Consistently, PSTVd infection in natural host potato also induced *Virp1* mRNA and increased promoter DNA methylation of certain endogenous genes.

Lacking protein-coding capacity, interference with DNA methylation by PSTVd must direct interaction of its genomic RNA or derivatives with host factors. It has recently been reported that infection of cucumber with a nuclear-replicating HSVd is accompanied by dynamic changes in DNA methylation of host ribosomal (rb)RNA genes, likely associated with alterations in the levels of endogenous rb-sRNAs^35^. However, host factors involved in changing DNA methylation have not been identified. Consistent with previous findings that the PSTVd interaction with Virp1 was required for PSTVd infection in both *Nicotiana* plants and protoplasts^32^,^33^, in this study, we demonstrate that silencing of *Virp1* by an RNAi construct prevented PSTVd replication in *N. benthamiana* plants. Virp1 is a bromodomain-containing protein with an atypical RNA binding domain and a nuclear localization signal. Bromodomain-containing proteins have the “chromatin association mark” and have been recognized as a new partnership for silencing^37^,^38^. For example, SWI/SNF is a well-characterized chromatin remodeling complex, and the Swi2/snf2 bromodomain is important for SWI/SNF-mediated displacement of acetylated histones in Yeast and Hela nucleosomes^39^,^40^. Two plant-specific subfamilies of SNF2 domain-containing proteins, CLSY1 and DRD1, are implicated in DNA methylation and nuclear RNA silencing, along with Pol IV and Pol V, respectively^41^,^42^,^43^. Moreover, DRD1 could contribute to the dynamic regulation of DNA methylation^42^, as Virp1 is also a SNF2-like subfamily protein and its carboxy-terminal RNA-binding domain has been shown to interact specifically with the right terminal domain of the viroid RNA *in vivo* and *in vitro*^32^,^33^. Our results show that PSTVd replication in both *N. benthamiana* and potato plants induced *Virp1* and enhanced gene silencing by interfering with DNA methylation. We propose that the Virp1 protein itself may play an additional role in RNA-mediated methylation, in addition to its requirement for PSTVd infection. Alternatively, PSTVd may possess the capability to interact with other SNF2 domain-containing protein(s), such as CLSY1 and DRD1, which are associated with nuclear RNA silencing. Therefore, PSTVd replication in the nucleus may interfere with the nuclear silencing pathway, resulting in the alteration of DNA methylation. In fact, the lower effect of Virp1 than PSTVd on DNA methylation suggests that other factors are involved in or affected by PSTVd replication and might also contribute to accelerating the silencing process. It has been reported that DNA-dependent RNA polymerase II (Pol II) is implicated in PSTVd transcription^28^,^29^,^30^,^31^, and that Pol II coordinates Pol IV and Pol V in TGS and RdDM in Arabidopsis^44^,^45^,^46^. Thus, subunits of Pol II that are implicated in PSTVd transcription could also be possible candidates involved in the alteration of DNA methylation during PSTVd infection. Our results provide new insights into the plant-viroid interaction on viroid pathogenicity by subverting the plant cell silencing machinery.

Viroid molecules are presumed to exist in their native state in the cell as rod-like structures, characterized by a series of short double helices and internal loops resulting from intramolecular base-pairing^27^,^47^, with a structural similarity to the host’s imperfect stem-loop miRNA precursors, which look similar to the terminal parts of viroids. Although microRNAs are known to control the expression of genes involved in several developmental processes^48^,^49^,^50^,^51^, our data showed that PSTVd infection was unlikely to affect the biosynthesis of endogenous miRNAs, even though potato plants infected with PSTVd showed alteration in leaf morphology and growth, consistent with the previous report that *Citrus exocortis viroid* (CEVd)-infected tomato did not affect the endogenous miRNA pathway^17^. In fact, it has been shown that an artificial miRNA that corresponds to sequences within the PSTVd virulence modulating region (VMR) induced abnormal phenotypes in *Nicotiana tabacum* and *N. benthamiana* that closely resemble those displayed by PSTVd-infected plants^52^, suggesting that PSTVd-derived siRNAs could direct RNA silencing of targeted host gene(s). This could also account for viroid pathogenicity^53^.

The PSTVd in its native state is an effective DCL substrate but is largely inaccessible by the RISC complex^26^. It is likely that the DCL-mediated primary viRNAs are essential but not sufficient for antiviral defense^14^. We presume that the increase in expression of *Virp1* upon PSTVd has a dual role in the PSTVd-host interaction. On the one hand, PSTVd replication requires Virp1. On the other hand, the RNA binding domain and nuclear localization signal of Virp1 facilitate viroid cutting into viRNAs by DCL proteins located in the nucleus. This may compensate for the inefficient RISC-associated anti-PSTVd in viroid replicating plant cells.

In summary, in this study, we demonstrate a novel activity of a subviral silencing enhancer in targeting DNA methylation associated with host Virp1, a bromodomain-containing protein. To the best of our knowledge, this is the first demonstration of involvement of a host factor in interfering with DNA methylation induced by a pathogenic non-coding viroid RNA. We cannot rule out that PSTVd replication would also cause reduction of host genome DNA methylation during infection, as recently reported for HSVd infection in cucumber for dynamic alteration of rbDNA^35^. Genome-wide analyses of the DNA methylome in PSTVd-infected plants would help to define the impact of PSTVd infection on DNA methylation and host gene transcription in viroid pathogenesis.

## Additional Information

**How to cite this article**: Lv, D.-Q. *et al*. Replication of a pathogenic non-coding RNA increases DNA methylation in plants associated with a bromodomain-containing viroid-binding protein. *Sci. Rep.* **6**, 35751; doi: 10.1038/srep35751 (2016).

## Supplementary Material

Supplementary Information
