# Supplementary material for: Replication of a pathogenic non-coding RNA increases DNA methylation in plants associated with a bromodomain-containing viroid-binding protein
Source: Sci Rep. 2016 Oct 21;6:35751. doi: 10.1038/srep35751 (PMC5073342; doi:10.1038/srep35751)

Replication of a pathogenic non-coding RNA increases DNA methylation in plants associated with a bromodomain-containing viroid-binding protein

Dian-Qiu Lv[1,2#], Shang-Wu Liu[1,3#], Jian-Hua Zhao[3], Bang-Jun Zhou[3†], Shao-Peng Wang[1], Hui-Shan Guo[3] and Yuan-Yuan Fang[3]*

[1]Virus-free Seedling Research Institute, Heilongjiang Academy of Agricultural Sciences, Harbin, China
[2]Heilongjiang Academy of Agricultural Sciences Postdoctoral Programme
[3]State Key Laboratory of Plant Genomics, Institute of Microbiology, Chinese Academy of Sciences, Beijing, China

[†]Current address: Plant Science Innovation Center and Plant Pathology department, University of Nebraska, Lincoln

[#]equal contribution

*Corresponding author: Yuan-Yuan Fang
e-mail: fangyy@im.ac.cn
Tel: 010-64854989
Fax: 010-64847989

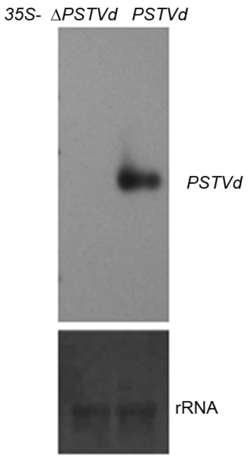

Supplementary Figure S1.

Confirmation of non-replicative 35S-ΔPSTVd and replicative 35S-PSTVd. 35S-ΔPSTVd and 35S-PSTVd were transient expressed in *Nicotiana benthamiana* via Agro-infiltration, total RNAs were isolated from infiltrated leaves, the *PSTVd* RNA was examined by RNA gel blotting analysis.

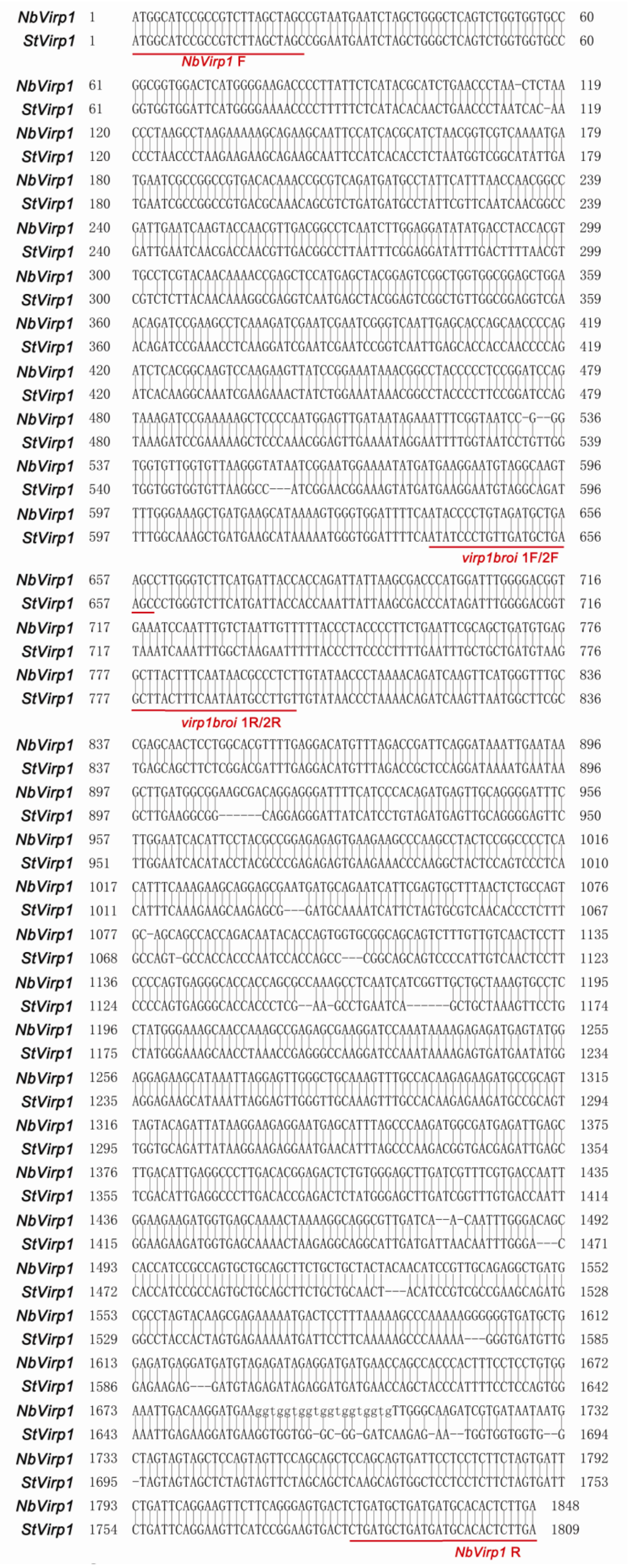
NbVirp1
StVirp1
NbVirp1 F
virp1broi 1F/2F
virp1broi 1R/2R
NbVirp1 R

Supplementary Figure S2

Alignment of *NbVirp1* and *StVirp1* sequences. The *NbVirp1* and *StVirp1* sequences share 87% identity at the nucleotide level. The primers for amplification of the full-length *NbVirp1* and generation of NbVirp1-RNAi constructs are marked at related positions in red.

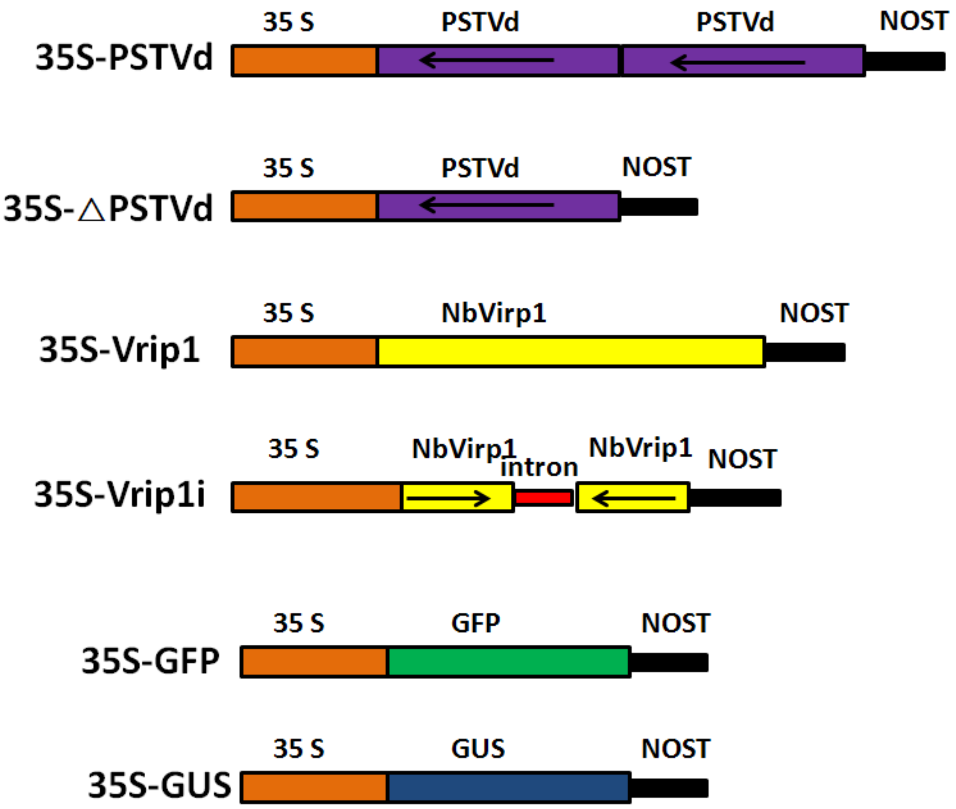

Supplementary Figure S3

A simple diagram showing all constructs used in this study.

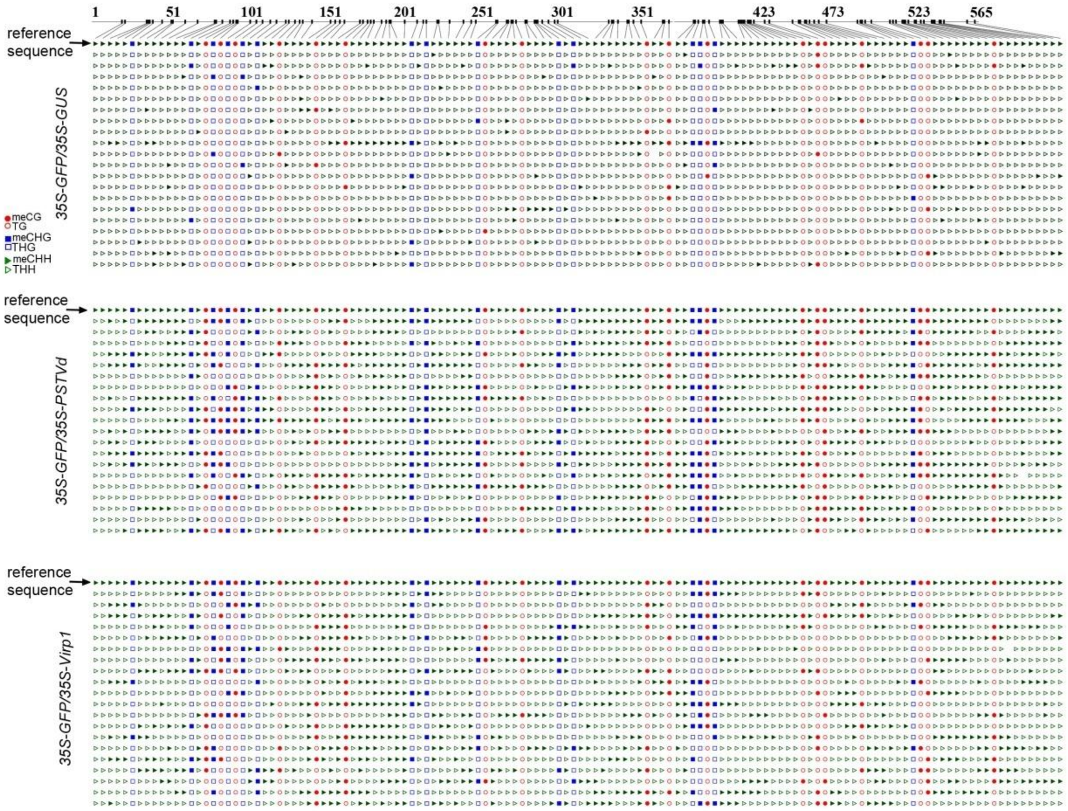

Supplementary Figure S4.

The original data of bisulfite sequencing of the 35S promoter of the *35S-GFP* transgene in 16c plants co-infiltrated with *35S-GFP*/*35S-GUS*, *35S-GFP*/*35S-PSTVd* or *35S-GFP*/*35S-Virp1*. Red cycle: CG site, blue square: CHG and green triangle: CHH. Methylated cytosines were symbolized with related solid shapes. Positions of cytosines are indicated at the top.

Supplementary Table S1. List of primer and probe used in this study

Primer name	Primer sequence(5'to3')	Purpose
PC0054	**GGATCCCTGAAGCGCTCCTCCGAGCCG**	For 35S-PSTVd, PSTVd probe
PH0055	**GAGCTCCCGGGAAACCTGGAGCGAACTGG**	production
NbVirp1 F	**CCCGGG**ATGGCATCCGCCGTCTTAGCTAGC	For 35S-Virp1, Virp1 probe
NbVirp1 R	ACGCGTTCAAGAGTGTGCATCATCAG	production
P8 F	AA**GAGCTC**ACCAACTGCGGTTCCAAG	For 35S-ΔPSTVd
P9 R	ATCTAGAGGTTCCTGTGGTTCACACC	
virp1broi1F	G**CTCGAGTCTAGA**ATATCCCAGTTGATGCTGAAGC	For Virp1i
virp1broi1R	C**AGATCT**ACAAGGCATTATTGAAAGTAAGC	
virp1broi2F	C**GGATCCGAGCTC**ATATCCCTGTTGATGCTGAAGC	
virp1broi2R	G**CTGCAG**ACAAGGCATTATTGAAAGTAAGC	
35S+G F	AAGTAATAGAGATTGGAGTTTTTAAAAAGGTAG	For DNA Bisulphite Sequencing
35S+G R	AATAAATTTTCCRTATATTACATCACCTTCACCCTC	
Q11752F	AGGGTGGAGATCGACTTA	Cellulose synthase primer for
Q11752R	ATGGGATTGAGATGTGATAAAC	qRT-PCR
Q14997F	ACTAATCTCATCATCGCC	Zinc finger protein primer for
Q14997R	CTCTGGATAGTAGTACATT	qRT-PCR
Q19517F	CATACCCAGAGGGACGAT	Chitin-binding lectin 1 primer for
Q19517R	CTTGACAGTTTACAGGAGC	qRT-PCR
Q35647F	AGGGCTCAAGGGATTTAC	Disease resistance response
Q35647R	CCTCCAACAATAGGCATC	protein primer for qRT-PCR
EF1-αF	ATTGGAAACGGATATGCTCCA	the internal standard EF1-a
EF1-αR	TCCTTACCTGAACGCCTGTCA	primer for qRT-PCR
11752F	AGTCTCAGTGTGGCATAGTAGGAAC	Cellulose synthase primer for
11752R	AGAGGGAGTATGAGTTTAGGGGTTG	McrBC-PCR
14997F	ACAACCAAATCTCCCTTCG	Zinc finger protein primer for
14997R	CTCTGATAAAGACGACCA	McrBC-PCR
19517F	ATCCCGAATAGGAGAAGT	Chitin-binding lectin 1 primer for
19517R	ATGCTGGTATGTTGTATTGA	McrBC-PCR
35647F	CACCCTTGCCTATGCTAC	Disease resistance response
35647R	GAGAATCGCTCAGTGTTG	protein primer for McrBC-PCR
GFP-F	ATGAGTAAAGGAGAAGAACTTTTC	For GFP probe production
GFP-R	TTTGTATAGTTCATCCATGC	
U6	GCTAATCTTCTCTGTATCGTTCC	For U6 detection
miR159	TAGAGCTCCCTTCAATCCAAA	For miR159 detection
miR167	TAGATCATGCTGGCAGCTTCA	For miR167 detection

Full-length figures

Figure 1A

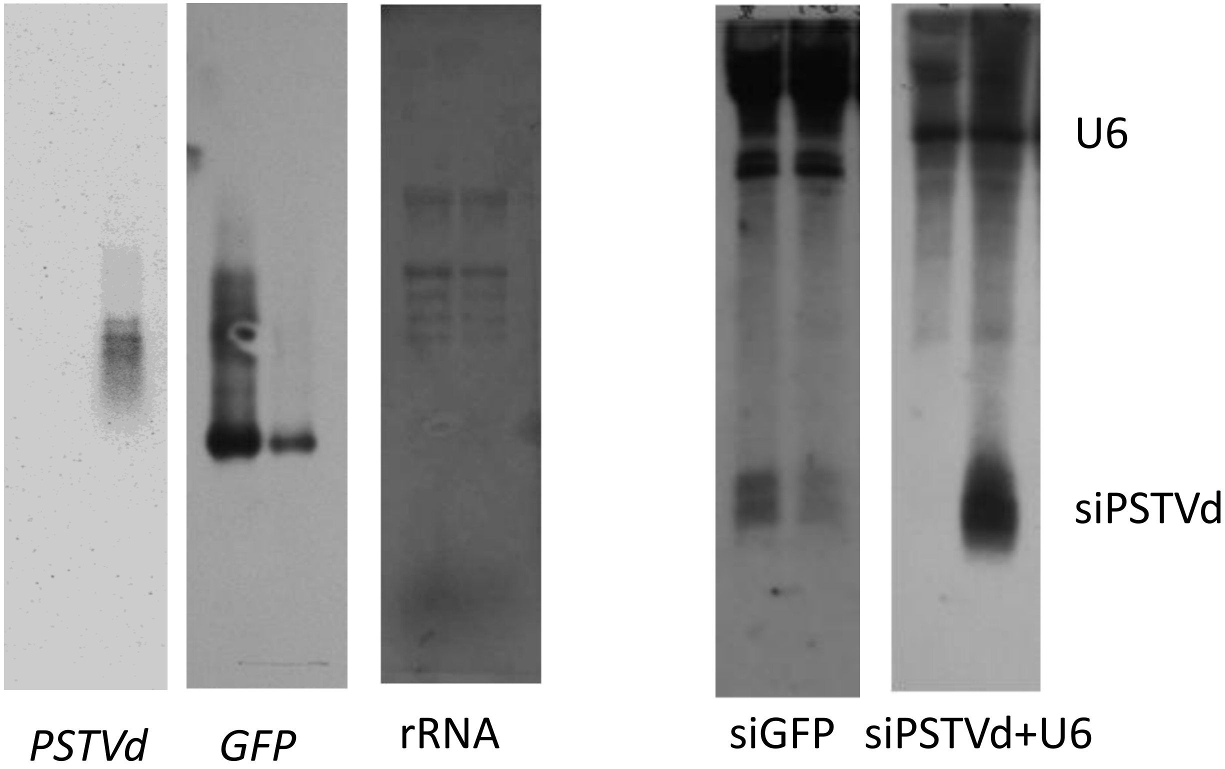

Figure 2B

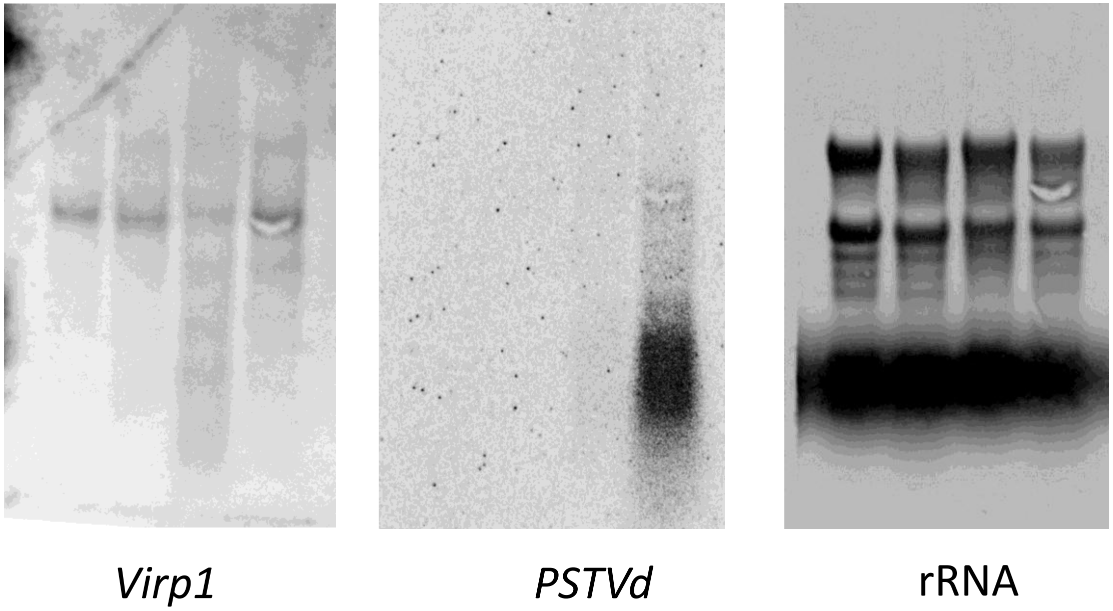

Figure 3B

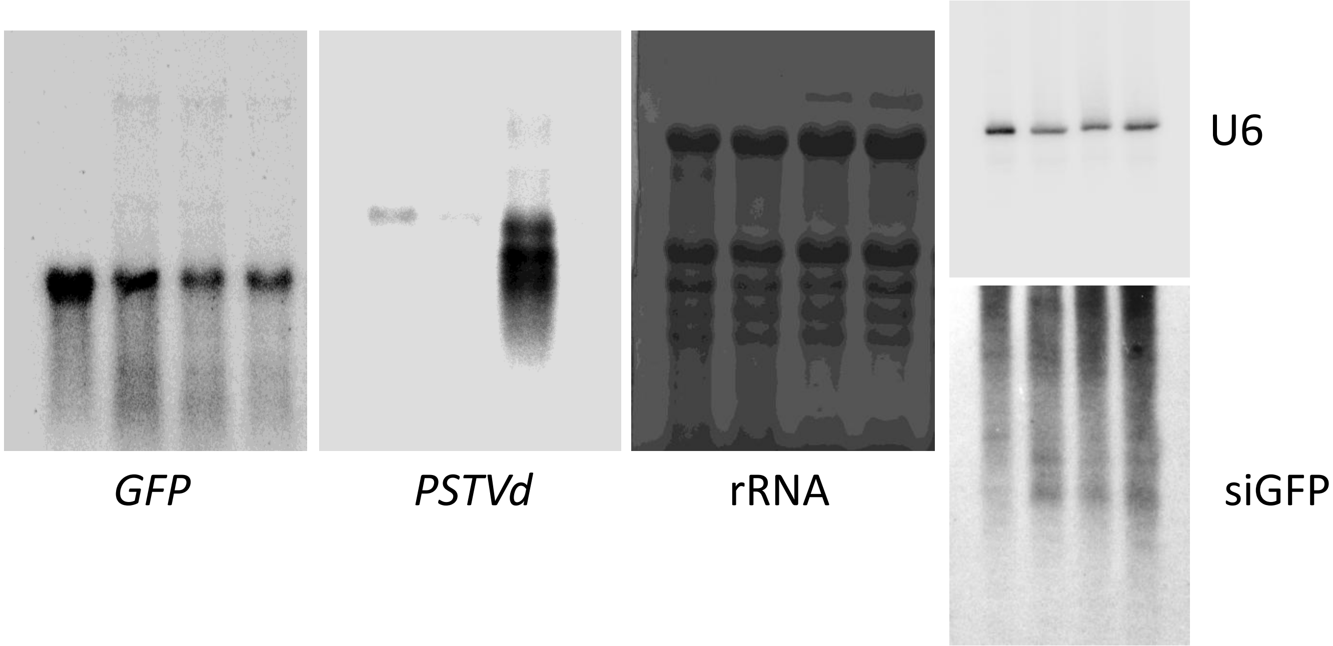

Figure 4B

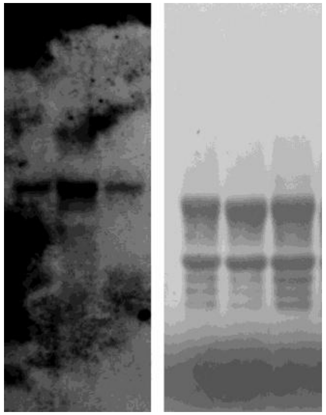

Figure 4C

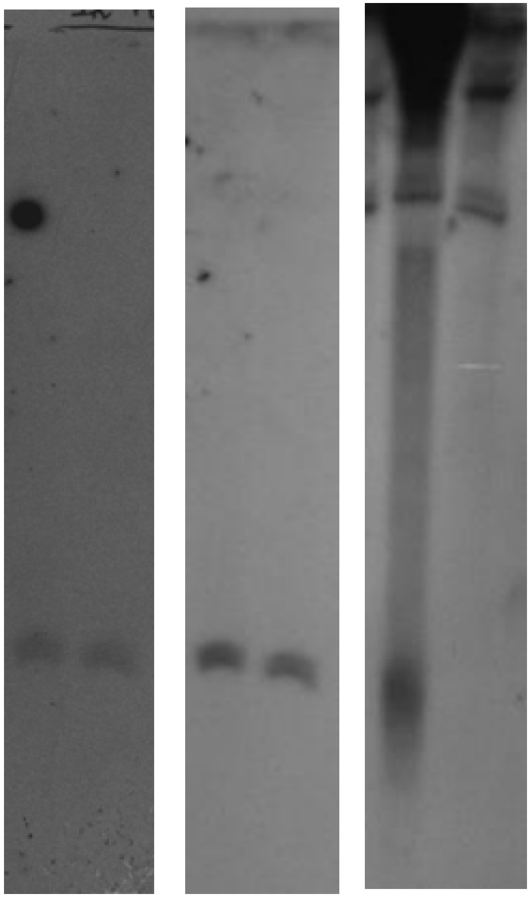

Figure 4D

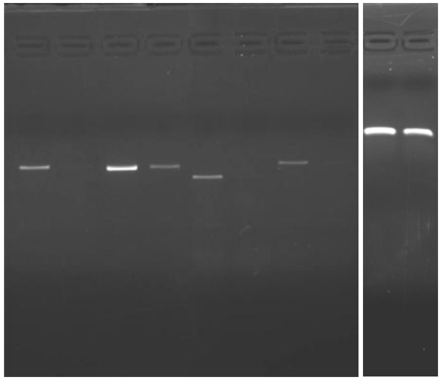

Supplement: Supplementary Information [file srep35751-s1.pdf]
